# Lasing in Live Mitotic and Non-Phagocytic Cells by Efficient Delivery of Microresonators

**DOI:** 10.1038/srep40877

**Published:** 2017-01-19

**Authors:** Marcel Schubert, Klara Volckaert, Markus Karl, Andrew Morton, Philipp Liehm, Gareth B. Miles, Simon J. Powis, Malte C. Gather

**Affiliations:** 1 SUPA, School of Physics and Astronomy, University of St Andrews, St Andrews KY16 9SS, United Kingdom; 2 School of Psychology and Neuroscience, University of St Andrews, St Andrews KY16 9SS, United Kingdom; 3 School of Medicine, University of St Andrews, St Andrews KY16 9SS, United Kingdom

## Abstract

Reliable methods to individually track large numbers of cells in real time are urgently needed to advance our understanding of important biological processes like cancer metastasis, neuronal network development and wound healing. It has recently been suggested to introduce microscopic whispering gallery mode lasers into the cytoplasm of cells and to use their characteristic, size-dependent emission spectrum as optical barcode but so far there is no evidence that this approach is generally applicable. Here, we describe a method that drastically improves intracellular delivery of resonators for several cell types, including mitotic and non-phagocytic cells. In addition, we characterize the influence of resonator size on the spectral characteristics of the emitted laser light and identify an optimum size range that facilitates tagging and tracking of thousands of cells simultaneously. Finally, we observe that the microresonators remain internalized by cells during cell division, which enables tagging several generations of cells.

The unique properties of laser light render lasers one of the most versatile tools in modern science, and in particular in the life sciences. Modern super-resolution microscopy^1^, optical tweezers^2^ and precise optogenetics^3^,^4^ would be inconceivable without the high intensity, long coherence and narrow spectral width that laser light provides. However, irrespective of the application, laser radiation so far has to be supplied by external sources, which is often obscured by strong scattering and absorption in biological tissue, thus posing limits for *in vivo* applications. Generating laser light directly within biological samples may provide a way to overcome this fundamental hurdle and in addition may lead to radically different ways of using laser emission in biological experiments^5^,^6^,^7^.

The development of complex organisms involves a dynamic interplay of large numbers of cells and many different cell types. Understanding such systems as a whole requires determining the properties of the constituent cell populations. Cell lineage tracing with single cell resolution in whole organisms has been recently achieved, either through advanced microscopy or by a combination of DNA barcodes and genome editing^8^,^9^,^10^. However, both approaches are extremely data intensive and either rely on fully transparent samples and continuous imaging, or are highly invasive requiring deep sequencing of DNA from all cells of interest. Studying long-term processes in tissue or changes of cellular composition in real time, e.g. during modulation and reshaping of biological tissue or in circulating tumor cell clusters, therefore calls for faster and more robust cell tags. Strategies to discriminate between different cells include spectral encoding based on fluorescent particles or proteins (introduced by endocytosis or through transgenic routes)^11^,^12^,^13^ and numerous barcoding techniques^14^,^15^. However, due to the spectrally broad emission of the fluorescent labels used in these, the number of distinguishable tags is relatively small. By contrast, spectral multiplexing of the intense and spectrally narrow emission of lasers could enable a dramatically larger number of tags but biointegration of lasers into cells remained elusive due to their complexity, size and sensitive optical components. Very recently this hurdle was overcome by introducing optically pumped microscopic whispering gallery mode (WGM) resonator-based lasers into live cells^16^,^17^. WGM resonators trap light by total internal reflection inside a high refractive index material and, when doped with a fluorescent dye, form highly versatile microscopic lasers^18^,^19^. The spectral position of the sharp laser lines generated by WGM lasers is very sensitive to resonator size and two proof-of-concept demonstrations have illustrated how this could be used for barcode type tagging and tracking of thousands of individual cells^16^,^17^.

Here, we demonstrate the practical feasibility of intracellular laser-based cell tracking by establishing routes for robust and efficient introduction of WGM resonators into a wide range of cells, including primary cells and cells from the nervous system, which are generally believed to be non-phagocytic. We quantify and optimize the efficiency of the intracellular delivery of our lasers and investigate the influence of resonator size on this process. (Efficient internalization of resonators with a substantial range of sizes is crucial to yield sufficient variability between the lasing spectra and thus allows discrimination between large numbers of cells.) Finally, we study the long-term fate of internalized resonators in mitotic cells. We find that the intracellular laser approach facilitates cell tagging over several generations of cells as resonators are retained even during cell division.

## Results

### Resonator internalization by various cell types

**Figure 1:**
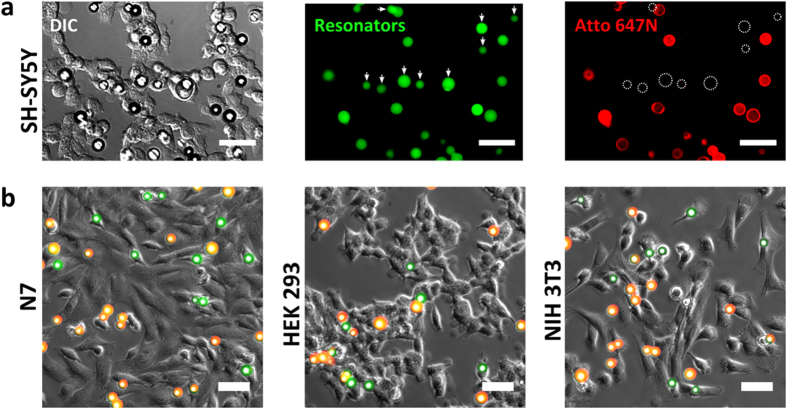
Internalization assay reveals resonator uptake by different cell types. **(a)** Representative images from a bioassay for quantification of resonator uptake efficiency in SH-SY5Y cells. DIC microscopy overview image (left), green bulk fluorescence of WGM resonators (center) and red fluorescence from surface staining by the cell-impermeable Atto 647N-streptavidin conjugate (right). White dashed circles and white arrows indicate the position of phagocytosed resonators. **(b)** Overlay of phase contrast (greyscale) with fluorescence microscopy images for N7, HEK 293 and NIH 3T3 cells. Phagocytosed resonators appear green while extracellular resonators appear yellow-orange due to the overlap of the bulk fluorescence (green channel) with the Atto 647 N surface staining (red channel). Scale bars: 50 μm.

The WGM resonators used in this study are formed by polystyrene spheres with a nominal average diameter of 15 μm (coefficient of variation, ~14%). The spheres are doped with a highly fluorescent green-emitting dye that serves as gain medium and facilitates laser emission upon optical excitation. To reliably assess uptake of our WGM resonators by cells, we modified a previously reported internalization assay and combined it with fluorescence microscopy^20^,^21^. The assay is based on a cell impermeable, red fluorescent streptavidin conjugate (Atto 647N-streptavidin) that stains the surface of non-phagocytosed biotinylated resonators but leaves internalized biotinylated resonators unstained. Figure 1a shows a representative example of the internalization assay, here performed on SH-SY5Y neuroblastoma cells. The WGM resonators are easily identified by their intense green fluorescence. In addition, any non-internalized resonators show clear red fluorescence which results from surface staining by the streptavidin conjugate. Conversely, resonators showing green but no red fluorescence can be confidently counted as intracellular. Overlaying these images with differential interference contrast (DIC) or phase contrast (PC) microscopy images allows direct evaluation of the phagocytic behaviour of different cell lines. Figure 1b shows further examples of the internalization assay for the neuronal cell line N7 as well as for HEK 293 and NIH 3T3 fibroblasts, clearly revealing the phagocytic capacity of all of these cell types.

### Quantifying and optimizing resonator uptake

**Figure 2:**
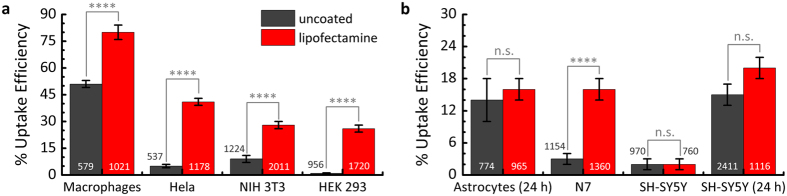
Uptake efficiency of uncoated (grey) and lipofectamine coated (red) WGM resonators for different cell types as determined by the internalization bioassay. **(a)** Primary human macrophages, epithelial (Hela), fibroblast (NIH 3T3) and HEK 293 cells. **(b)** Cells from the nervous system, including primary mouse astrocytes and neuronal cell lines N7 and SH-SY5Y. Unless indicated otherwise, uptake efficiencies were determined after 4 h of incubation. Error bars represent standard error of the mean. Two sample t-test is used to evaluate statistical significance (****p < 0.0001; n.s. = not significant). The number at each column indicates the number of resonators analyzed.

Due to its robust and efficient read-out, our uptake assay can analyze large numbers of uptake events and can thus also be used to obtain a quantitative measure of uptake efficiency. Here, we investigated a broad range of different cell types, including primary macrophages and astrocytes as well as the cell lines Hela, HEK 293, NIH 3T3, N7 and SH-SY5Y and performed an internalization assay of 500 to 2,500 resonators for each cell type. As proliferation rates between different cell types vary, with primary cells often not dividing at all, phagocytosed resonators were counted relatively soon after adding them to the cell culture dish (4 h incubation time) to avoid large changes in the total number of cells. Dividing the number of internalized resonators by the total number of resonators in each field of view yielded the uptake efficiency for each cell type (Fig. 2a). To place the uptake efficiency of the cells under investigation in context, phagocytic macrophages were used as a reference. Macrophages phagocytose large objects efficiently^22^ and the primary human macrophages tested here internalized 51% of the resonators within just 4 h of incubation. Uptake efficiencies for the other cell types were lower by comparison, ranging from 9% for fibroblasts (NIH 3T3) to only 0.9% for HEK 293 cells. We also investigated cells from the central nervous system, into which foreign objects are known to be difficult to introduce. Indeed uptake efficiencies were only 3% and 2% for the neuronal cell lines N7 and SH-SY5Y, respectively (Fig. 2b). However, upon increasing the incubation time from 4 to 24 hours, we observed a steep increase in uptake efficiency from 2% to 16% for SH-SY5Y. A similar efficiency of 14% after 24 h was found for primary mouse astrocytes, which are less migratory and therefore may require more time to make initial contact with the resonators.

Although these uptake efficiencies are significant, they remain considerably below the values achieved for macrophages and a further improvement will be desirable to efficiently tag cells in large populations. In the literature, different liposome coatings have been reported to assist with the uptake of much smaller polymer microspheres (about 2 μm in size) by non-phagocytic cells^21^,^23^,^24^. Liposomes consist of a formulation of neutral and positively charged lipid molecules that can facilitate contact and fusion with negatively charged cell membranes. We hypothesized that opsonizing our resonators with a liposome-forming transfection reagent (lipofectamine) may allow resonators to enter cells more efficiently. Under optimized coating conditions (Supplementary Fig. 1 and Supplementary Fig. 2), we indeed observed a strongly increased uptake efficiency for the majority of investigated cells. In macrophages about 80% of the resonators were internalized and for the cell lines Hela, NIH 3T3 and HEK 293 uptake efficiencies reached 41%, 28% and 26% after 4 h, respectively. The increase in uptake efficiency upon lipofectamine treatment was statistically significant for all four cell types. The achieved uptake efficiency values are comparable to the transfection efficiencies commonly achieved during liposome-based injection of much smaller plasmid DNA particles into cells. A strong increase in uptake efficiency, to 16%, was also observed for N7 whereas no significant influence of the lipofectamine coating was seen for mouse astrocytes and SH-SY5Y. We conclude that surface coating and increased incubation time or a combination thereof are suitable methods to achieve high tagging efficiencies in a variety of cell types, even though the intracellular recognition and processing of the resonators by the cells may differ between the two approaches^21^,^24^,^25^,^26^. (We did not find any indication that the different approaches for laser internalization affect the spectral properties of the lasers.)

### Effect of resonator size on phagocytosis

**Figure 3:**
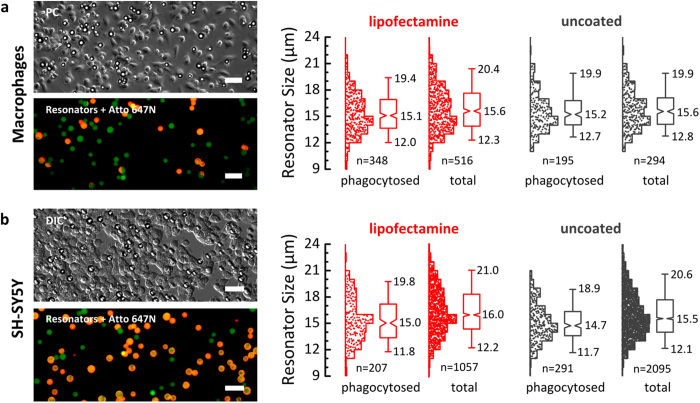
Size dependence of resonator uptake. **(a)** Primary human macrophages and **(b)** SH-SY5Y cells. Left: Representative microscopy images of uptake experiments. Right: Histograms and statistical analysis of resonator size for phagocytosed resonators and for the entire resonator sample (total = phagocytosed + external). *n* indicates number of resonators for each histogram. Box plots show lower and upper quartile, and notches represent 95% confidence interval for the median at the center of the notch. Whiskers extend to 5^th^ and 95^th^ percentile (Altman style). Median size and sizes at 5%/95% percentile are listed. Scale bars: 50 μm.

We further investigated if the resonator size influences the uptake efficiency. Successful internalization of large foreign objects generally involves the redistribution of actin structures within the cell to induce membrane closure. This process has been reported to depend strongly on the shape but less on the size of the objects^22^,^27^,^28^. However, particle size has been found to affect the ability for internalization if the particle volume is comparable to the volume of the cell; in fact the volume of the cell has been assumed to be the upper limit for the phagocytic capability of macrophages^22^. Figure 3 shows the size distribution of four samples of between 300 and 2,000 resonators which were used for further uptake experiments with macrophages and SH-SY5Y cells, both with and without lipofectamine treatment. Generally, the difference between the size distribution of phagocytosed resonators and the total resonator size distribtion was small, with the median resonator size varying between 14.7 and 16.0 μm in all cases (close to the nominal average size of 15 μm). The majority of resonators (90%) have sizes between around 12 and 20 μm. For macrophages incubated with uncoated resonators, statistical testing (non-parametric Mann-Whitney test) found no significant difference between the size distribution of phagocytozed resonators and the total size distribution (i.e. phagocytosed plus external resonators). For the lipofectamine-coated sample, the size of the phagocytosed resonators is smaller by a statstically significant amount (Mann-Whitney test, p < 0.01) even though the difference in median value is only 0.5 μm. This suggests that the resonator uptake by macrophages is almost independent of their size, at least for diameters between 10 and 20 μm. For SH-SY5Y, the difference between the total and phagocytozed size distribution was again small but slightly more pronounced than in macrophages, with the median resonator size of uncoated and lipofectamine treated phagocytosed resonators being 0.8 μm and 1 μm smaller, respectively, than the median of the whole sample (Mann-Whitney test, p < 0.01). Moreover, there were almost no phagocytosed resonators larger than 20 μm and the size distributions of the phagocytozed resonators appear skewed above 15 μm when compared to the other samples. This suggests that the phagocytic capacity of SH-SY5Y saturates at resonator diameters of about 15 μm, which still represents a remarkable size for non-phagocytic neuronal-like cells. While the details of the process driving the phagocytosis of the large resonators are not fully understood, the weak size dependence represents an important finding for the application of biolasers to cell tagging (see below).

### Resonator size determines spectral characteristics of intracellular lasers

**Figure 4:**
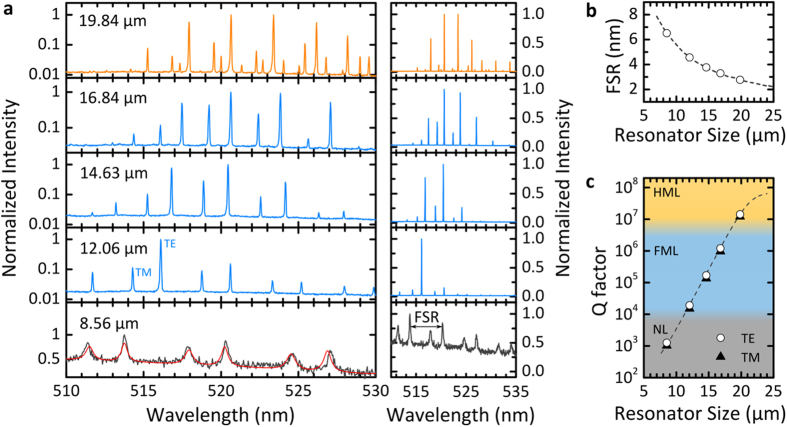
Effect of resonator size on lasing characteristics. **(a)** Comparison of emission spectra of resonators phagocytosed by SH-SY5Y cells on a logarithmic intensity scale (left) and linear scale (right). The resonator diameter is indicated for each spectrum. For the 8.56 μm resonator no lasing is observed and the experimentally observed (grey line) and simulated (red line) linewidths increase drastically. **(b)** Simulated free spectral range (FSR) and **(c)** Q factor (dashed lines) as function of resonator diameter. Symbols mark the resonator diameters given in (a). In the simulation, refractive indices of *n*_PS_ = 1.6 and *n*_cell_ = 1.37 were assumed for the resonator and the cell, respectively^16^,^31^.

The confinement of light and the emission spectrum of a WGM resonator depend strongly on its size. In practice, distinguishing one resonator from another requires determining the position of its lasing peaks with high precision and we have previously estimated that with the distribution of resonator sizes used above, about 2,000 cells can be unambiguously identified^16^. To investigate the influence of resonator size in more detail, we recorded the lasing spectra of a number of WGM resonators with different diameters that were located inside SH-SY5Y cells. Lasing was achieved by excitation with a diode-pumped pulsed 473 nm laser. Figure 4 shows the spectra for five different resonators with diameters covering the entire size range found in our resonator sample (*cf.* Fig. 3). The spectra were used to determine the size of the resonators by fitting the mode positions to the theoretical resonance wavelengths known from Mie theory^29^,^30^.

For a resonator with a diameter of about 12 μm – substantially smaller than the average resonator diameter in our sample – the lasing spectrum was composed of a characteristic regular pattern of narrow emission lines (Fig. 4a). These lines correspond to first radial order transverse electric (TE) and transverse magnetic (TM) radial modes. [For TE (TM) modes, the electric field oscillates parallel (perpendicular) and the magnetic field perpendicular (parallel) to the resonator surface.] A characteristic feature of this WGM spectrum is the spectral separation of adjacent TE modes, described as the free spectral range (FSR), which decreases with increasing resonator size (FSR 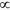 (diameter)^−1^ with R^2^ = 0.9997; Fig. 4b). The position of the dominant lasing mode and the FSR both depend strongly on resonator size and can be used as cell tag as we have previously proposed^16^. Light trapping is more effective in larger resonators due to the reduced surface curvature and this is reflected by an increase in the predicted quality factor (Q factor) of the resonator (Fig. 4c). For the 12 μm resonator we estimate a Q factor of 10^4^ (Fig. 4c). For larger resonators, Q factors approach 10^5^ and lasing spectra no longer comprise only one dominating TE mode but two or more modes of comparable intensity. In addition, the dominant peak tends to shift to longer wavelengths, as shown in Fig. 4a for the 14.63 μm and 16.84 μm diameter resonators. We attribute both effects to the improved confinement of the mode in larger diameter resonators which results in an increase in modal gain at longer wavelengths and increased self-absorption at shorter wavelengths. We find that the pump conditions (i.e., pump power, position of the pump spot, and polarization) can affect the relative intensity of the peaks associated with different modes, but not their spectral positions. Therefore, relative peak intensity may vary over time and thus is less useful for gathering information about cells. As the resonator diameter approaches 20 μm, a further change occurs. Besides the first radial mode order, higher order radial modes now also start to lase. This renders identification and assignment of the modes more difficult and would increase the complexity of cell tagging. Given that cellular uptake is also somewhat less efficient for these very large diameter resonators, we conclude that resonators with a size larger than 20 μm are less suitable for cell tagging experiments.

For resonators significantly smaller than 10 μm, we did not observe lasing. This is a result of their substantially lower Q factors. However, spectral peaks associated with WGMs were still visible in the spontaneous emission spectrum of these small resonators. Compared to the laser emission of the larger resonators, the emission peaks in the spontaneous fluorescence regime had a drastically increased line width (500 pm *vs.* 50 pm) and showed much poorer signal to fluorescence background ratio (1:1 *vs.* 100:1). This strongly reduces the number of individual cell tags that can be discriminated and means that multiple excitation pulses are required to acquire a clear spectrum. Therefore, use of these WGM resonators is less attractive, in particular for applications that require high throughput, e.g. flow environments, where the large signal of the lasing modes is a key advantage.

The three size-dependent operation regimes for intracellular WGM resonators – non-lasing (NL), first-order mode lasing (FML) and higher-order mode lasing (HML) – are summarized in Fig. 4c. We conclude that the optimum diameter for polystyrene-based spherical WGM laser tags lies between 10 and 20 μm.

### Long-term tracking in mitotic fibroblasts

The practicability of intracellular lasers for cell tagging relies critically on whether cells can keep resonators internalized over prolonged periods of time. In our previous work, we demonstrated continuous tracking of a group of primary human macrophages over 22 h^16^. While primary macrophages do not proliferate in culture, many cell types will undergo mitosis during extended experiments. This involves complex structural reorganizations within the cell and the question arises if the presence of the intracellular resonator impairs cell division. Furthermore, the lipofectamine coating used to aid phagocytosis may re-open cell membranes at a later stage and thus could cause cells to lose resonators.

**Figure 5:**
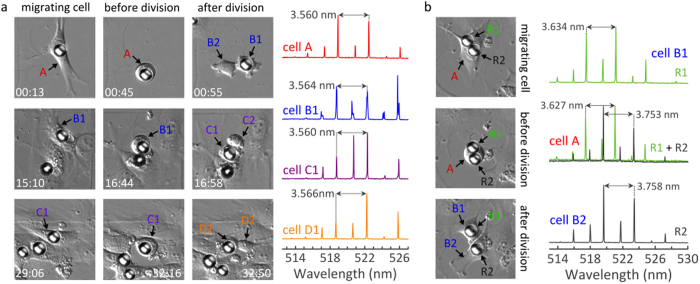
Long-term tracking of 3T3 fibroblasts over several cell generations. Mother cells are denoted as A (red) and subsequent daughter generations are labeled with B (blue), C (violet) and D (orange), respectively. **(a)** Left: DIC images of WGM laser within migrating cell, before, during and after three cycles of cell division. The times indicated in the images are in h:min and represent the time after acquisition of the first lasing spectrum. Right: Corresponding lasing spectra of the WGM resonator recorded during migratory period, i.e. between cell divisions. Arrows mark FSR between two neighboring TE modes. **(b)** Left: Tagging of both daughter cells (B1 and B2) from a mother cell carrying two intracellular lasers (R1 and R2). Right: Lasing spectra of resonators inside the mother cell (center, recorded separately for each resonator but plotted together) and after cell division (top/bottom). All DIC images show an area of 100 × 100 μm^2^.

To address these questions, we performed long-term tracking experiments over several days in mitotic 3T3 fibroblasts. The experiments started with cultures of low cell density (below 5% confluency). To increase the probability for resonator uptake under these conditions, lipofectamine-coated resonators were used. Figure 5a shows a 3T3 fibroblast cell shortly after resonator uptake. Over the course of the experiment, the cell followed a phenotypical migration pattern and underwent three cycles of cell division. The resonator was found to remain completely enclosed by the cell throughout the entire experiment, i.e. during both migration and division (Supplementary Video 1). In addition, the spectral emission pattern can be used to unambiguously identify the resonator. This is demonstrated by comparing the free spectral range at different time points. Between the first and last lasing experiment, i.e. over the course of 41 h, the FSR was found to vary by only 6 pm (Fig. 5a). The cell tagging capability can be extended further if more than one resonator is taken up by a cell. Figure 5b shows a 3T3 cell with two phagocytosed resonators which cannot be distinguished by microscopy because their sizes are too similar. However, when excited separately, each resonator showed a characteristic lasing mode pattern. After cell division each of the two daughter cells carried a single resonator and by comparing the FSR each of them can be tracked separately. More examples of single (Supplementary Fig. 3) and double (Supplementary Fig. 4) resonator tagged cells are displayed in the Supporting Information. A total of 30 divisions were observed for cells with a single resonator and six cell divisions were monitored for cells with two internalized resonators. For all of these, the resonators were transferred to their subsequent daughter cells, and five of the six cells with two resonators divided in a 1:1 ratio while in one case both resonators remained within one of the two daughter cells. The largest change in FSR that we observed in these experiments was 17 pm. Due to the high intensity of the WGM laser emission, tracking can be performed by using just a single 2 ns pump pulse for each WGM laser (Supplementary Fig. 5). Finally, we note that by the end of the experiment some resonators had been exposed to about 5,000 excitation pulses, without noticeable reduction in laser performance which shows the excellent stability of our microlasers. Supplementary Fig. 6 provides data for prolonged excitation of a resonator with 10,000 pump pulses, showing that even under these extreme conditions the resonators maintain their excellent spectral characteristics, with the FSR varying by only 2 pm.

## Discussion

Delivery of microresonators into live cells is the fundamental step for any application of intracellular lasers. The experiments described here demonstrate the practical feasability of WGM microlaser-based cell tracking by revealing broad compatibility with cells of different type and origin. For Hela and astrocytes, uptake of small microspheres with sizes ranging from 1 to 3 μm was reported previously^21^,^23^,^32^,^33^. However, here we have shown that with proper surface functionalization these cells can readily internalize WGM resonators with volumes 300-fold larger than this, thus revealing a surprising ability of cells to internalize large polymer spheres. We also demonstrated an increase in the dimensions of phagocytosed objects for macrophages, with earlier reports showing uptake of spherical objects with diameters of up to 12.5 μm^22^,^26^ and eliptical disks of up to 18 μm × 7 μm × 4 μm^22^,^34^. Importantly, our study shows that microresonator internalization is highly feasible for all cell types investigated here, including cells generally understood to be non-phagocytic, like HEK 293, N7, and SH-SY5Y. To the best of our knowledge, no study has previously assessed the phagocytic uptake of such large objects in such a diverse range of cell types. Moreover, we identified incubation times and liposome surface treatment conditions that significantly improve uptake efficiency. The latter is particularly attractive for future *in vivo* applications given that there are a number of clinically approved liposomal formulations^35^. We expect that, in combination with flow cytometry and cell sorting, complete tagging of large cell populations will be readily achievable^24^,^36^.

Our combined analysis of the effect of resonator size on internalization and on lasing characteristics indicates that for polystyrene based WGM resonators diameters between 10 and 20 μm are ideal. Over this size range the uptake efficiency is largely independent of resonator size and pure first order radial mode lasing within living cells is observed which facilitates reliable cell tracking over several generations of cell division by measuring the FSR of the laser emission. To unambiguously label a maximum number of cells, the entire 10 to 20 μm size range should be used for future cell tagging experiments.

In addition to efficient internalization, long-term cell tracking requires robust retention of resonators, especially after liposome-assisted phagocytic delivery. Our experiments show that phagocytosed resonators remain internalized over days, even during cell division. This provides further evidence that the presence of resonators does not interfere with cell function, consistent with other reports on non-specific cellular recognition of objects with dimensions larger than about 1 μm. In fact, a recent study on the phagocytosis of silica spheres by bone derived macrophages finds that lysosomal destabilization-induced apoptosis is much more pronounced for sub-micron sized particles than for particles in the 3–10 μm size range^26^.

If further miniaturization of resonators is required for certain applications, this would be best achieved through use of spherical microspheres made of high refractive index glass^37^ or ceramics^17^,^38^. By introducing several small resonators into each cell, intracellular lasers could be used for lineage tracing of differentiated cells, e.g. to link the fate of a specific daughter cell to the cellular properties or structural environment of several preceding generations of mother cells. Another possible application is the systematic investigation of cell-cell interactions, e.g. the dynamic formation and fragmentation of tissue or cell agglomerates from heterogeneous cell mixtures, which will help to understand the composition and development of circulating tumor cell clusters. Beyond this, the ability to tag and track large numbers of individual cells for extended periods of time is likely to inspire a plethora of further new experiments that we cannot foresee today.

## Methods

### Cell Culture

HEK 293, Hela, NIH 3T3 and N7 cells were cultured in DMEM medium with 10 vol% fetal bovine serum (FBS) and 1 vol% penicillin-streptomycin (PS). Primary macrophages were generated after ethical review and informed consent from plastic adherent monocytes isolated from blood of normal healthy donors and were treated with 40 ng/ml granulocyte macrophage colony-stimulating factor (GM-CSF) in RPMI medium with 10 vol% FBS and 1 vol% PS. SH-SY5Y cells were cultured in DMEM medium supplemented with 15 vol% FBS, 1 vol% PS and 1 vol% MEM non-essential amino acids. Astrocytes were isolated from the cerebral cortex of postnatal day 2 mice following a previously described procedure^39^ and cultured in DMEM medium supplemented with 5 vol% FBS, 5 vol% horse serum and 1 vol% PS.

### Preparation of lipofectamine and biotin coated resonators

PS-DVB microspheres that were internally stained with a green fluorescent dye (15 μm, Fischer Scientific) were submersed in NHS-LC-LC-Biotin (10 mg/ml in DMSO) for 30 min to achieve non-covalent biotin coating. Subsequently, the resonators were washed five times with DI water and the resonator concentration was adjusted to 5 × 10^6^ ml^−1^. For liposome assisted phagocytosis, Lipofectamine 3000 (10 μl/ml in DI water, Thermo Fischer Scientific) was added to the resonators and left for 30 min. Afterwards, the resonators were washed once to remove excess lipofectamine.

### Internalization assay

2 × 10^5^ cells were seeded in two 35 mm dishes (one for the control, one for the lipofectamine coated resonators) the day before the assay using the corresponding medium. Cells were washed with PBS and 2 × 10^5^ resonators in 1 mL of medium were added. Unless noted otherwise, the dishes were incubated for 4 h. Following incubation, resonators were stained with 10 μl ATTO 647N-streptavidin (1 mg/ml) and incubated for 15 min. Prior to imaging, the cells were washed and fixed in 4% formaldehyde in PBS for 15 minutes.

Resonator internalization was observed with an inverted optical fluorescence microscope using phase contrast, green fluorescence and red fluorescence to visualize internalization. Ten fields of view were taken for each dish corresponding to a range of 500 to 2500 resonators being analyzed. Resonators of the green and red fluorescence images were counted using a Fiji macro which analyzed any particle larger than 400 pixels (about 8 μm). Experiments on NIH 3T3 and HEK 293 were performed as triplicates while for all other cell types a single experiment was performed. The mean average uptake efficiency and the size distribution includes all fields of view. To extract resonator size distributions from the intrinsic fluorescence images of the resonators, these were first thresholded and then analyzed with the ImageJ function “Analyze Particles”.

### Intracellular lasing

Cells were seeded in glass bottom petri dishes with imprinted 500 μm grid (Ibidi μ-dish Grid-500) and mounted on an inverted microscope equipped with a stage-top microscope incubator (which allowed control of temperature, humidity and provided 5% CO_2_ atmosphere). DIC time-lapse were captured at 10x magnification with a frame every 2 minutes. Illumination was switched off between subsequent frames. A Q-switched and mode-locked diode laser with a wavelength, pulse width and repetition rate of 473 nm, 2 ns and 100 Hz, respectively, was used as pump source. A pump energy of 10 nJ per pulse was used. Excitation of the intracellular lasers and collection of the emission was through the same microscope objective and the diameter of the pump beam in the focal plane was approximately 20 μm. The excitation spot was manually aligned onto individual resonators which takes approximately 5–10 s per resonator. For long-term experiments, each resonator was measured up to 7 times, i.e. accumulating a total exposure of 3500 to 7000 pulses which resulted in no detectable degradation. Emission from the resonators was passed through a dichroic mirror and focused onto a 303 mm spectrograph coupled to a cooled CCD camera (Andor) equipped with a 1800 lines mm^−1^ grating. Experimental FSR values were determined by fitting Gaussian peaks to the WGM lasing peaks of two dominant TE modes.

The emission spectra below threshold were calculated according to a previously reported method^40^. Q factors and FSR were calculated from simulated spectra by computing the total average power radiated from a uniform distribution of fluorophores confined in a dielectric sphere (extension of classical Mie theory)^30^.

### Data availability

The research data supporting this publication can be accessed at http://dx.doi. org/10.17630/ec246742-c392-41f8-865d-31ad86801a63.

## Additional Information

**How to cite this article**: Schubert, M. *et al*. Lasing in Live Mitotic and Non-Phagocytic Cells by Efficient Delivery of Microresonators. *Sci. Rep.* **7**, 40877; doi: 10.1038/srep40877 (2017).

**Publisher's note:** Springer Nature remains neutral with regard to jurisdictional claims in published maps and institutional affiliations.

## Supplementary Material

Supplementary Video 1

Supplementary Information
