# Supplementary material for: Lasing in Live Mitotic and Non-Phagocytic Cells by Efficient Delivery of Microresonators
Source: Sci Rep. 2017 Jan 19;7:40877. doi: 10.1038/srep40877 (PMC5244359; doi:10.1038/srep40877)
Supplement: Supplementary Information [file srep40877-s2.pdf]

Supplementary Information

Lasing in Live Mitotic and Non-Phagocytic Cells by Efficient Delivery of Microresonators

Marcel Schubert[1], Klara Volckaert[1], Markus Karl[1], Andrew Morton[1], Philipp Liehm[1], Gareth B. Miles[2], Simon J. Powis[3], Malte C. Gather*[1]

[1] SUPA, School of Physics and Astronomy, University of St Andrews, St Andrews KY16 9SS, United Kingdom
[2] School of Psychology and Neuroscience, University of St Andrews, St Andrews KY16 9SS, United Kingdom
[3] School of Medicine, University of St Andrews, St Andrews KY16 9SS, United Kingdom

*Corresponding Author: mcg6@st-andrews.ac.uk

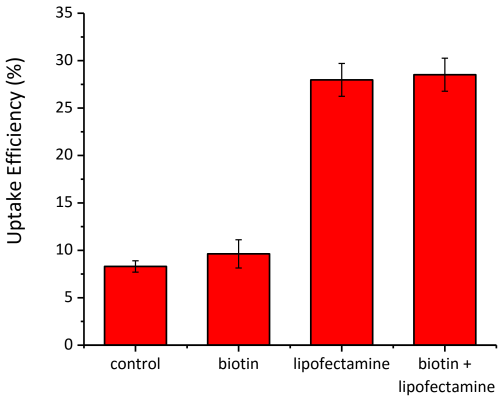

Supplementary Figure 1 │ Uptake efficiency in NIH 3T3 cells for different surface modifications of the polymer resonators. No effect on uptake efficiency is seen for opsonizing the beads with biotin compared to uncoated control beads. However, a strong increase in the uptake is observed when resonators are coated with lipofectamine where again no effect of the biotin is observed.

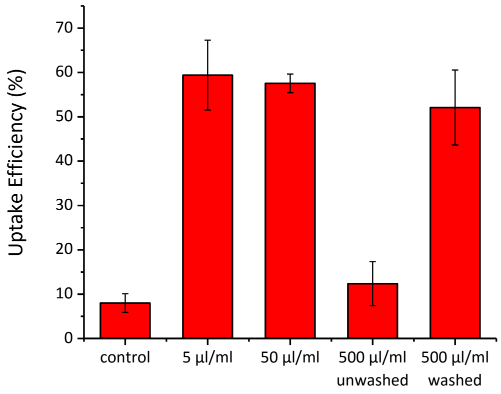

Supplementary Figure 2 │ Effect of lipofectamine concentration on resonator uptake investigated for HEK 293 cells. The strongly reduced efficiency for the 500 µl/ml concentration is due to lipofectamine-induced apoptosis. However, if resonators are washed, no cytotoxic effect was observed and a similar uptake efficiency as for the lower concentrations was observed.

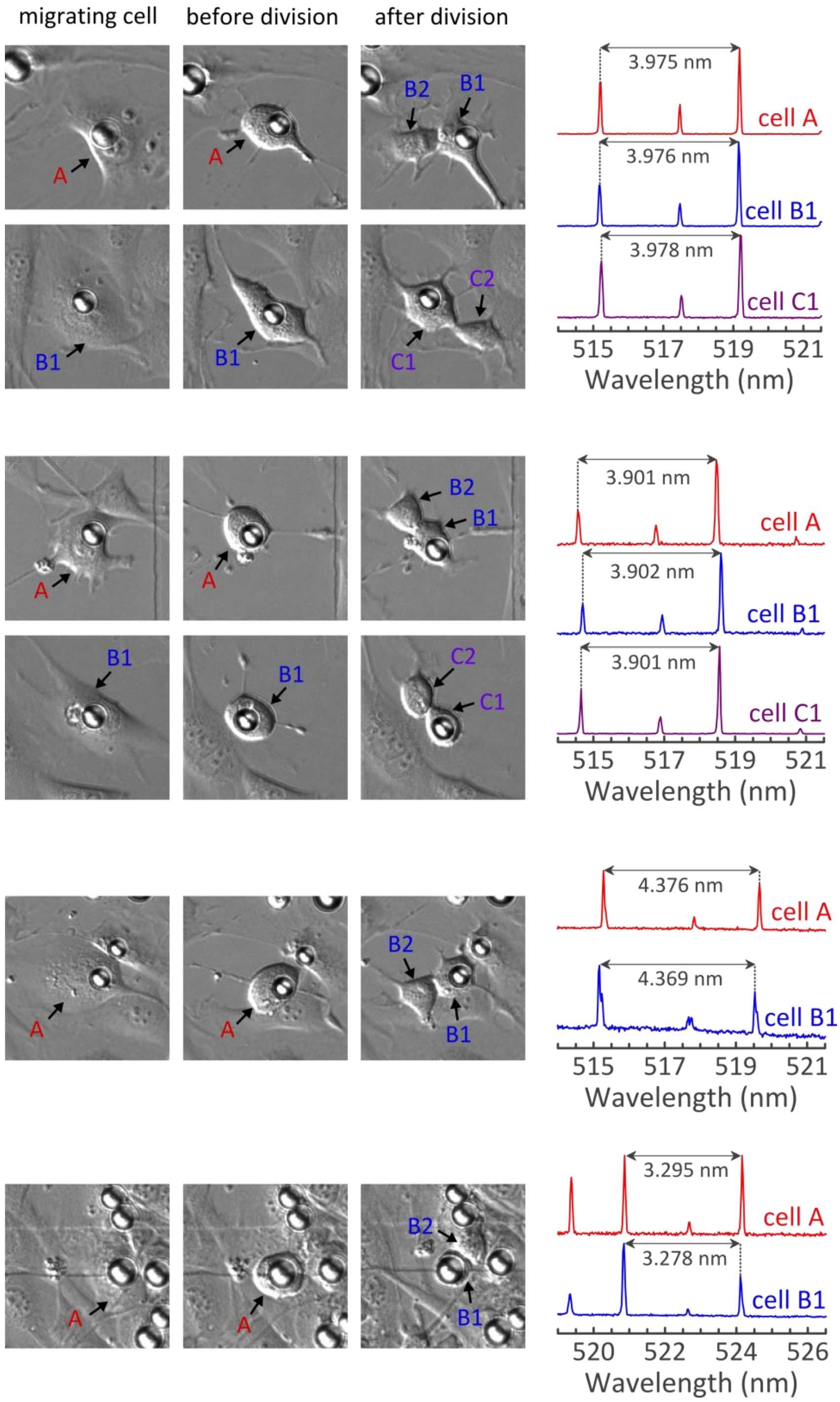

Supplementary Figure 3 Long-term tracking of NIH 3T3 fibroblasts tagged each by a single WGM resonator. Mother cells are denoted as A (red) and subsequent daughter generations are labeled with B (blue), and C (violet), respectively. Left: DIC images of intracellular laser within the migrating cell, before cell division and after cell division. Right: Corresponding lasing spectra of the WGM resonator recorded during cell migration. Arrows mark FSR between two neighboring TE modes given for each subsequent cell generation. All DIC images show an area of 100 x 100 µm^2.

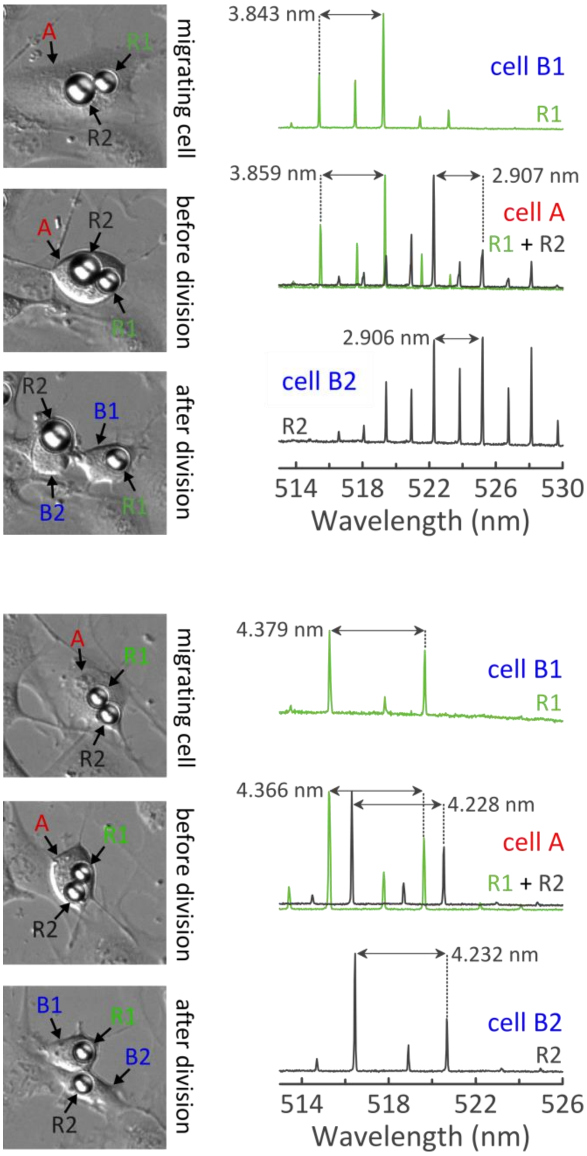

Supplementary Figure 4 Left: Tagging of both daughter cells (B1 and B2) from a mother NIH 3T3 cell carrying two intracellular lasers (R1 and R2). Right: Lasing spectra of resonators inside the mother cell (center, recorded separately for each resonator but plotted together) and after cell division (top/bottom) with arrows marking the FSR between two neighboring TE modes. All DIC images show an area of 100 x 100 µm^2.

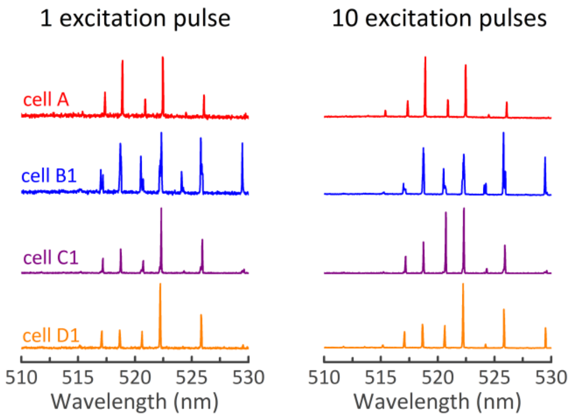

Supplementary Figure 5 Comparison of single excitation pulse spectra (left) with spectra that have been averaged over 10 excitation pulses (right). For all spectra several TM and TE modes can be identified which allows the extraction of the FSR that is then used to tag individual cells. All spectra are normalized to their maximum and displayed on a linear scale. Changes in the relative intensity of different modes between the spectra acquired with 1 and 10 excitation pulses are due to slightly altered pump conditions between the two independent measurements.

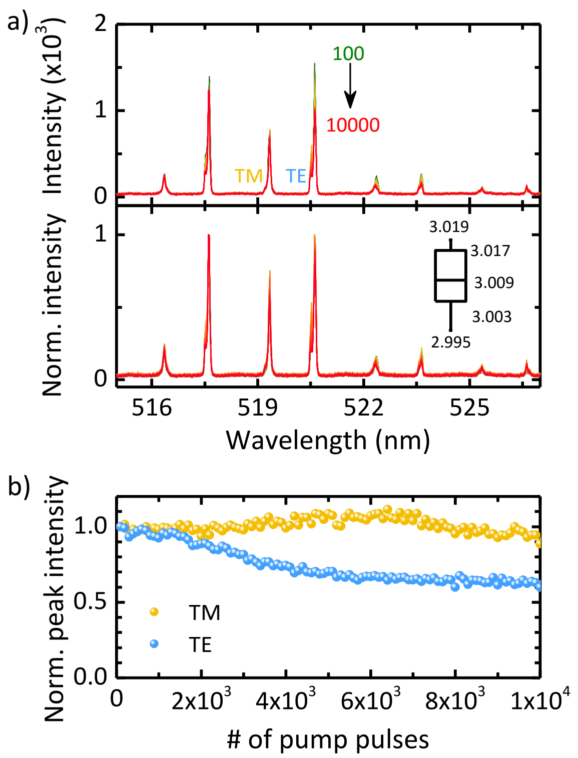

Supplementary Figure 6 a) Lasing spectra (top) and normalized lasing spectra (bottom) for an intracellular resonator in a fixed cell sample under continuous pump excitation. Integration time for each spectrum was 1 s and the repetition frequency of the excitation was 100 Hz. The arrow indicates a slight decrease in intensity for a lasing WGM between the first and last spectrum, with the number of pump pulses indicated. The box plot represents the statistical analysis of the extracted FSR were the center line, box margins and whiskers mark the mean, the upper and lower quartile, and the minimum and maximum FSR. **b)** Change of intensity of lasing peak for a TM (yellow) and TE (blue) mode under continuous pump excitation. The two investigated modes are labeled in a) and the intensity was normalized to the intensity at the beginning of the measurement. Time-dependent mode competition is clearly visible. Nevertheless, the FSR can be determined precisely to a value of (3.008 ± 0.002) nm (mean ± standard error of the mean).

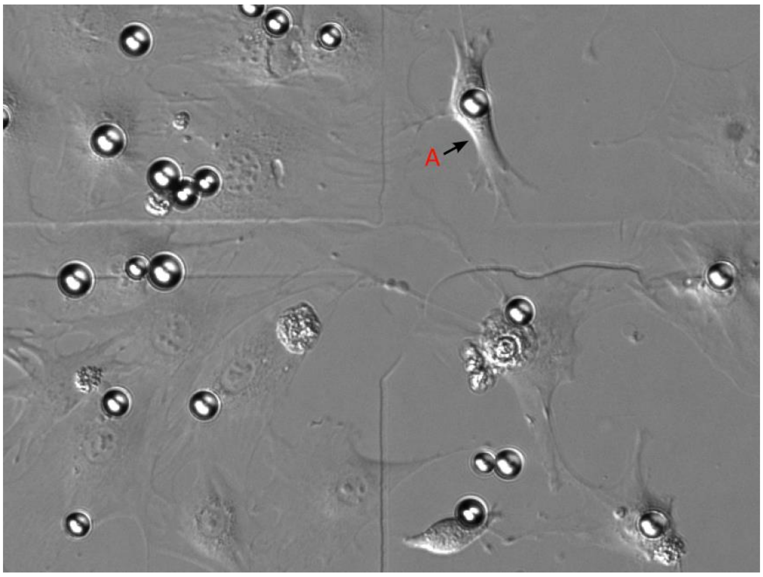

Supplementary Video 1 Time lapse DIC microscopy of 3 subsequent cell divisions of NIH 3T3 cells labelled with a single resonator (the resonator is in the upper right quadrant at the beginning of the video). Also shown is the division of a cell with two internalized resonators.
